# Supplementary material for: Chloride Applied via Fertilizer Affects Plant Nutrition and Coffee Quality
Source: Plants (Basel). 2023 Feb 15;12(4):885. doi: 10.3390/plants12040885 (PMC9968041; doi:10.3390/plants12040885)
Supplement: Supplementary file 1 [file plants-12-00885-s001.zip › plants-2150197-supplementary.pdf]

Table S1. Variables analyzed in the coffee beans for the harvest of 2017/2018

Treatments	CQ	Pol	TS	Caf	PPO	TTA	EC
T1	80.5a	6.3a	9.9a	1.06a	48.2a	183.2a	87.5a
T2	81.2a	6.4a	9.5a	1.04a	45.8a	182.8a	89.1a
T3	81.2a	6.3a	10.0a	1.03a	49.3a	191.5a	96.1a
T4	81.0a	6.5a	10.2a	1.08a	47.0a	193.3a	92.1a
T5	81.2a	6.4a	9.2a	1.05a	46.9a	185.2a	93.3a
Control	81.0a	6.7a	9.6a	1.00a	48.2a	181.7a	92.6a
CV (%)	1.7	2.3	5.5	6.5	3.3	4.3	4.6
Mean	81.0	6.4	9.7	1.04	47.6	186.5	91.8

CV (%) = coefficient of variation; CQ = cup quality score; Pol = total phenolic compounds (%); TS = content of total sugars (%); Caf = content of caffeine (%); PPO = polyphenol oxidase activity (u min^{-1} g^{-1}); TTA = total tritable acidity (mL NaOH 0.1 N 100 g^{-1}). Means followed by the same letter in column do not differ according to Tukey's test ($P > 0.05$). T1: 100% KCl; T2: 75% KCl + 25% K_2SO_4; T3: 50% KCl + 50% K_2SO_4; T4: 25% KCl + 75% K_2SO_4; T5: 100% K_2SO_4; control did not receive K_2O.

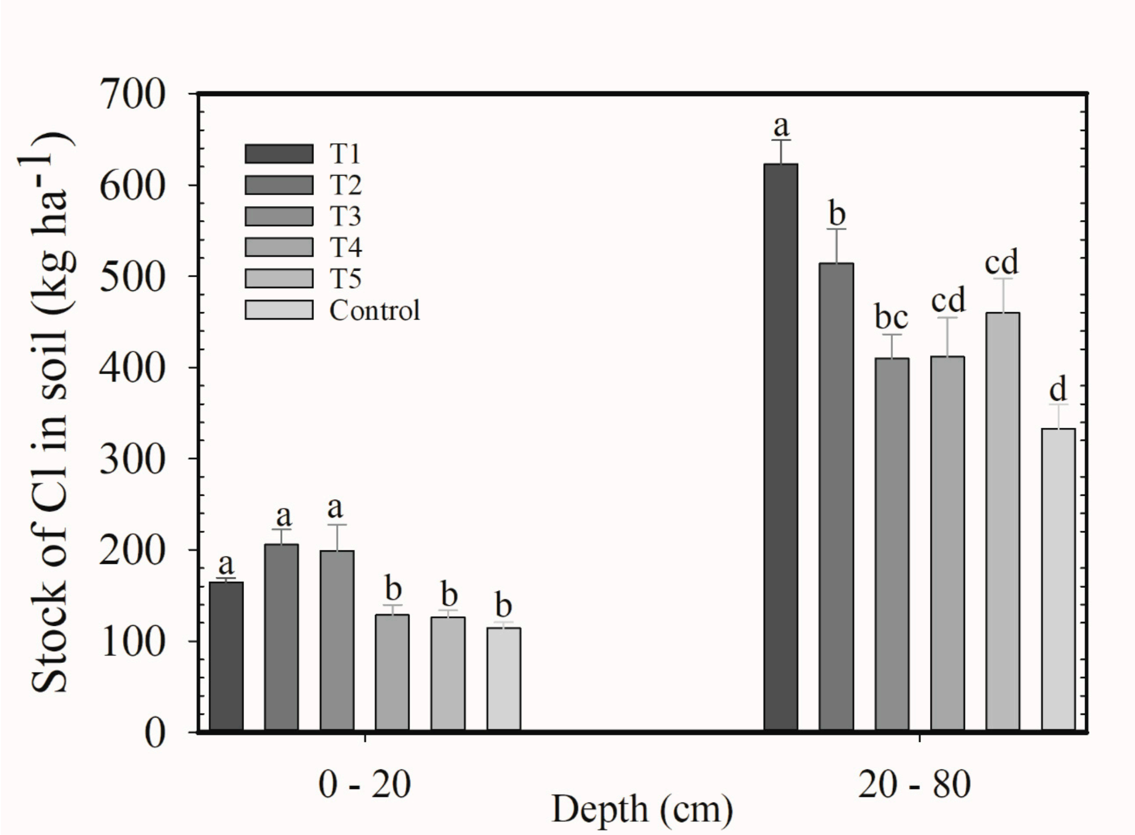

Figure S1. Stocks of Cl in the 0-20 and 20-80 cm layers after application of KCl and K_2SO_4 blends as cover fertilization on coffee plants. Harvest of 2017/2018. Means followed by the same letter do not differ according to Tukey's test ($P < 0.05$). Vertical bars indicate the standard error of the mean (n = 4). T1: 100% KCl; T2: 75% KCl + 25% K_2SO_4; T3: 50% KCl + 50% K_2SO_4; T4: 25% KCl + 75% K_2SO_4; T5: 100% K_2SO_4; control did not receive K_2O.

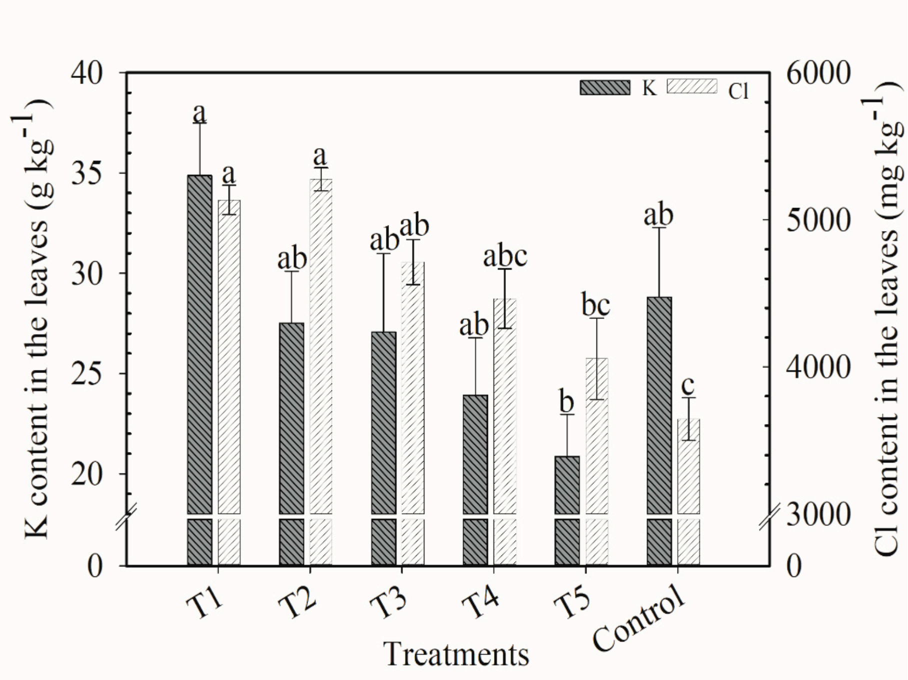

Figure S2. K and Cl content in the leaves of coffee plants, 20 days after application of the second cover fertilization parcel. Harvest of 2017/2018. Means followed by the same letter do not differ according to Tukey's test ($P < 0.05$). Vertical bars indicate the standard error of the mean ($n = 4$). T1: 100% KCl; T2: 75% KCl + 25% K_2SO_4; T3: 50% KCl + 50% K_2SO_4; T4: 25% KCl + 75% K_2SO_4; T5: 100% K_2SO_4; control did not receive K_2O.

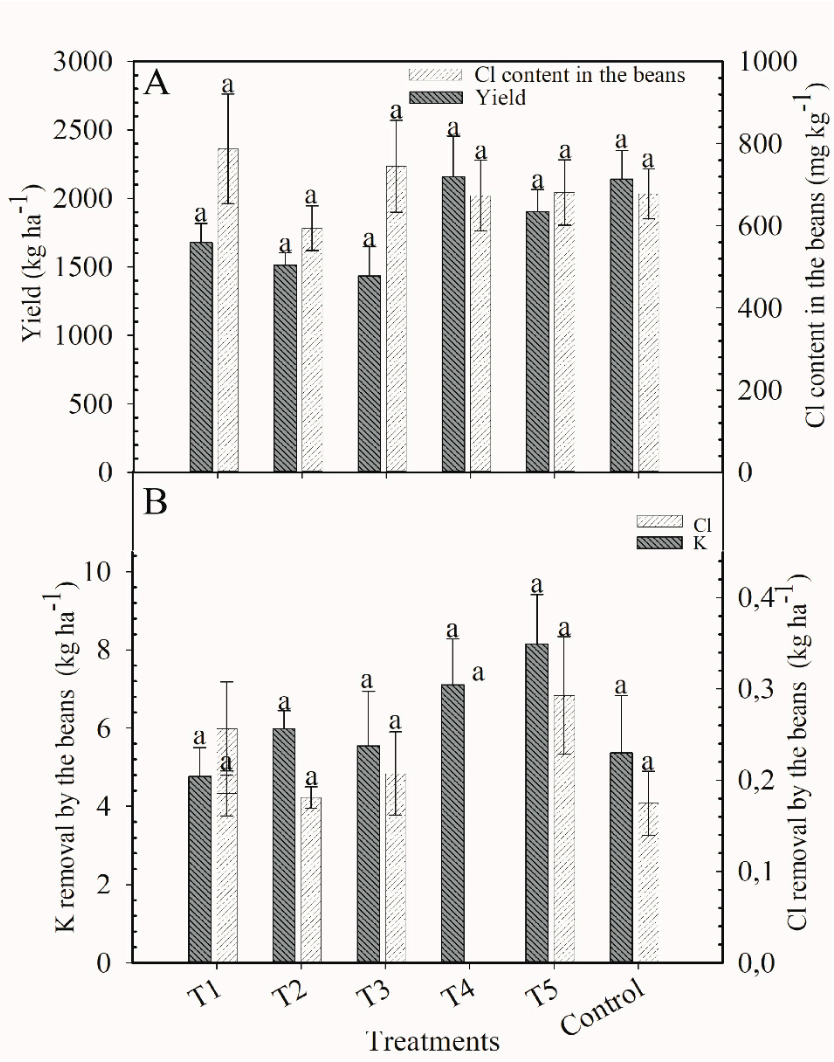

Figure S3. Yield of coffee plants and Cl content in the beans at cherry stage (A) and K and Cl re-moval by the beans (B) after application of blends of KCl e K_2SO_4 as cover fertilization. Harvest of 2017/2018. Means followed by the same letter do not differ according to Tukey's test ($P < 0.05$). Vertical bars indicate the standard error of the mean ($n = 4$). T1: 100% KCl; T2: 75% KCl + 25% K_2SO_4; T3: 50% KCl + 50% K_2SO_4; T4: 25% KCl + 75% K_2SO_4; T5: 100% K_2SO_4; control did not receive K_2O.

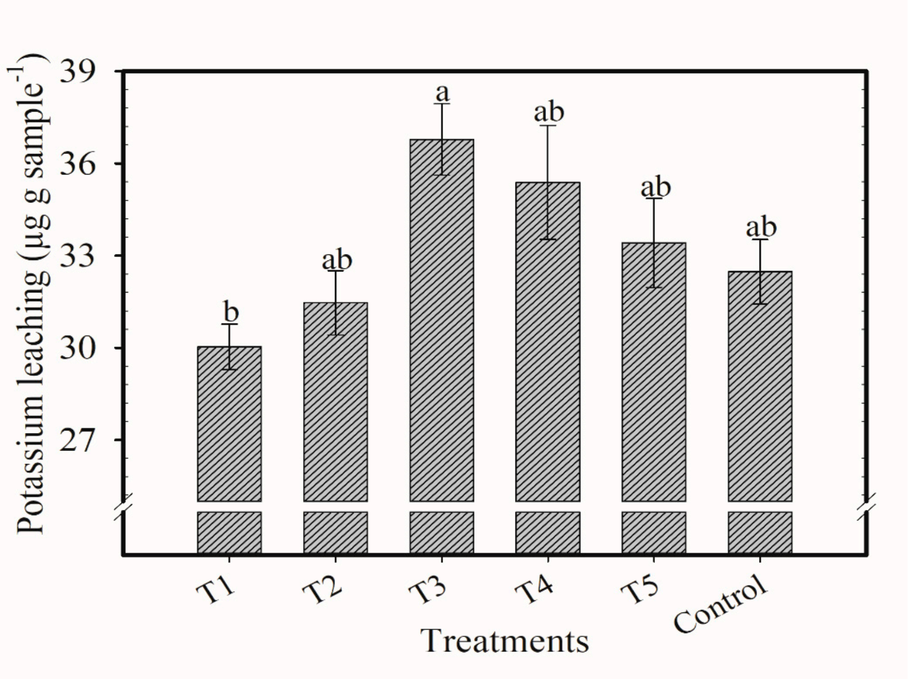

Figure S4. Potassium leaching in coffee beans at stage of cherry after application of blends of KCl e K_2SO_4 as cover fertilization. Harvest of 2017/2018. Means followed by the same letter do not differ according to Tukey's test ($P < 0.05$). Vertical bars indicate the standard error of the mean ($n = 4$). T1: 100% KCl; T2: 75% KCl + 25% K_2SO_4; T3: 50% KCl + 50% K_2SO_4; T4: 25% KCl + 75% K_2SO_4; T5: 100% K_2SO_4; control did not receive K_2O.

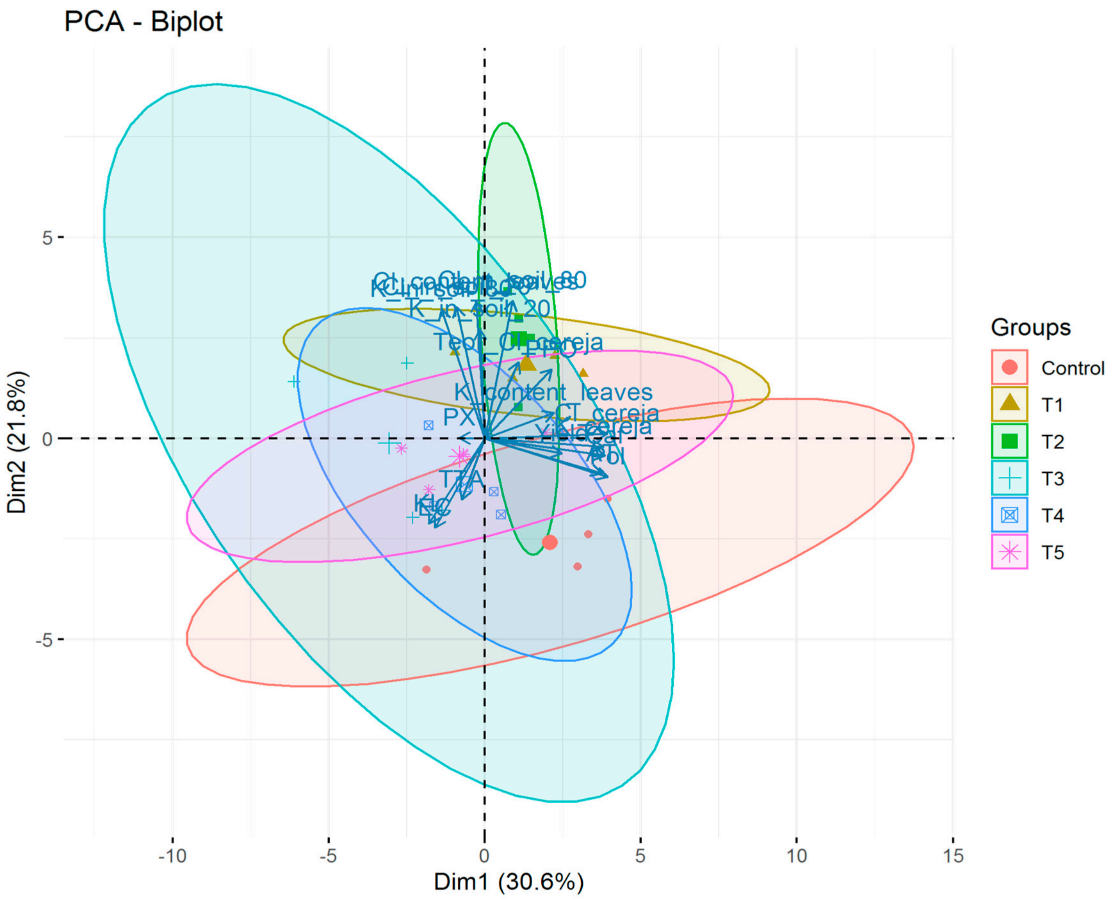

Figure S5. Principal component analysis for the harvest of 2017/2018 considering agronomic data. PPO = activity of the enzyme polyphenol oxidase; KL = K leaching; TTA = total titratable acidity; EC = electric conductivity; Pol = total phenolic compounds; Caf = content of caffeine; TS = content of total sugars; K in the soil 20 = stock of K in the 0-20 cm layer; K in the soil 80 = stock of K in the 20-80 cm layer; Cl in the soil 20 = stock of Cl in the 0-20 cm layer; Cl in the soil 80 = stock of Cl in the 20-80 cm layer, K rem. by beans: K removal by the beans, Cl rem. by beans: Cl removal by the beans. T1: 100% KCl; T2: 75% KCl + 25% K_2SO_4; T3: 50% KCl + 50% K_2SO_4; T4: 25% KCl + 75% K_2SO_4; T5: 100% K_2SO_4; control did not receive K_2O.

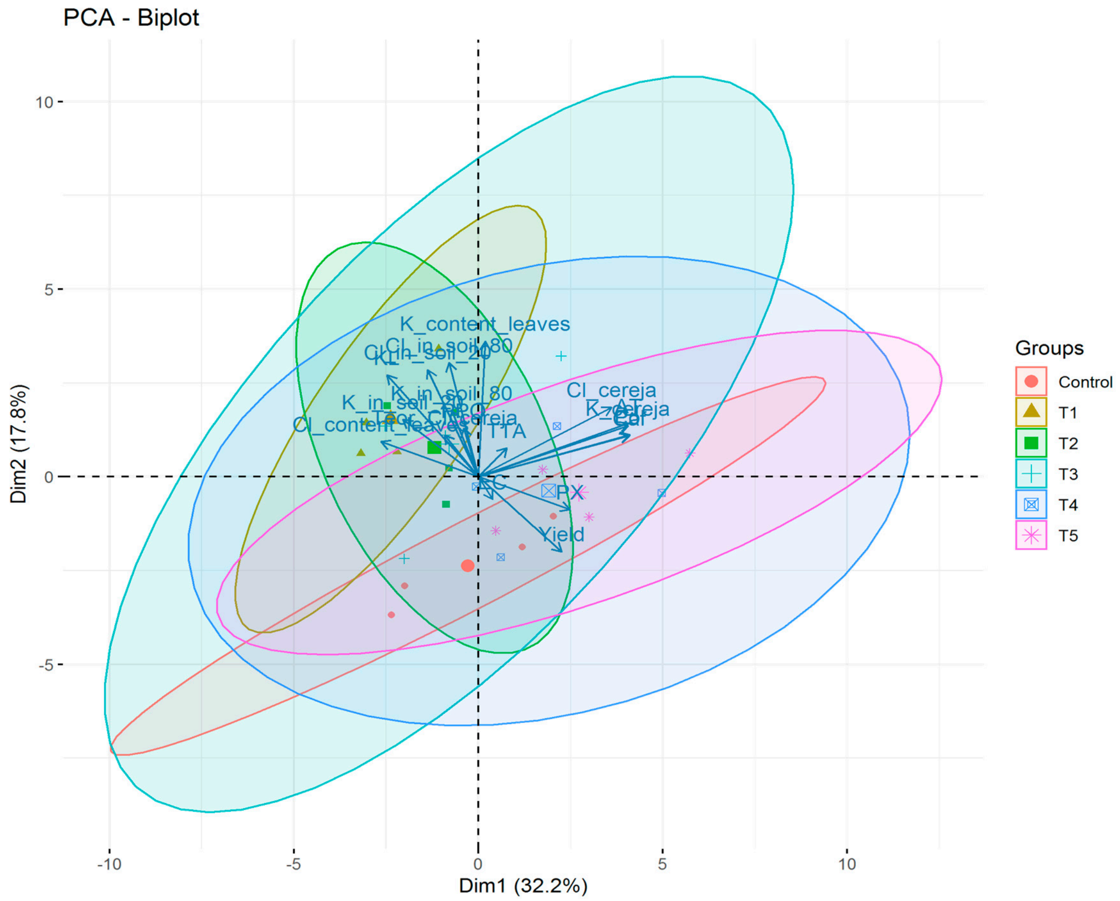

Figure S6. Principal component analysis for the harvest of 2018/2019 considering agronomic data. PPO = activity of the enzyme polyphenol oxidase; KL = K leaching; TTA = total titratable acidity; EC = electric conductivity; Pol = total phenolic compounds; Caf = content of caffeine; TS = content of total sugars; K in the soil 20 = stock of K in the 0-20 cm layer; K in the soil 80 = stock of K in the 20-80 cm layer; Cl in the soil 20 = stock of Cl in the 0-20 cm layer; Cl in the soil 80 = stock of Cl in the 20-80 cm layer, K rem. by beans: K removal by the beans, Cl rem. by beans: Cl removal by the beans. T1: 100% KCl; T2: 75% KCl + 25% K_2SO_4; T3: 50% KCl + 50% K_2SO_4; T4: 25% KCl + 75% K_2SO_4; T5: 100% K_2SO_4; control did not receive K_2O.

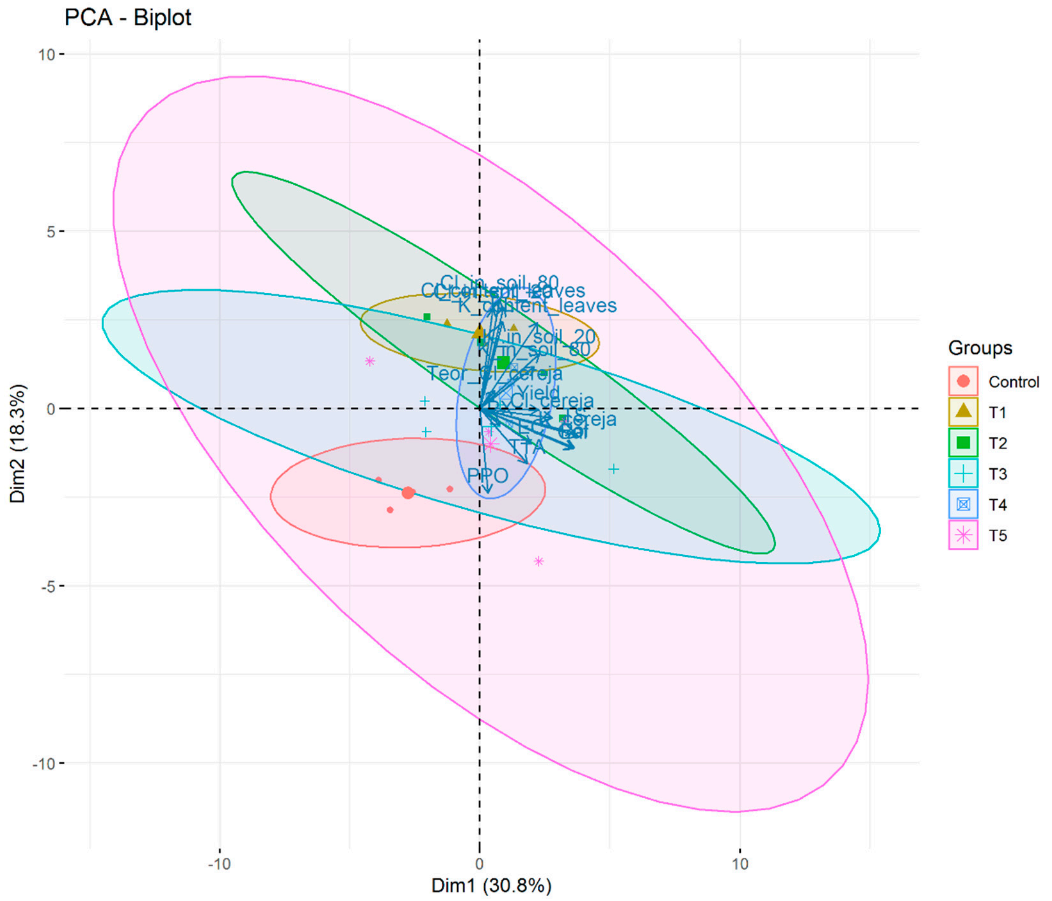

Figure S7. Principal component analysis for the harvest of 2019/2020 considering agronomic data. PPO = activity of the enzyme polyphenol oxidase; KL = K leaching; TTA = total titratable acidity; EC = electric conductivity; Pol = total phenolic compounds; Caf = content of caffeine; TS = content of total sugars; K in the soil 20 = stock of K in the 0-20 cm layer; K in the soil 80 = stock of K in the 20-80 cm layer; Cl in the soil 20 = stock of Cl in the 0-20 cm layer; Cl in the soil 80 = stock of Cl in the 20-80 cm layer, K rem. by beans: K removal by the beans, Cl rem. by beans: Cl removal by the beans. T1: 100% KCl; T2: 75% KCl + 25% K_2SO_4; T3: 50% KCl + 50% K_2SO_4; T4: 25% KCl + 75% K_2SO_4; T5: 100% K_2SO_4; control did not receive K_2O.

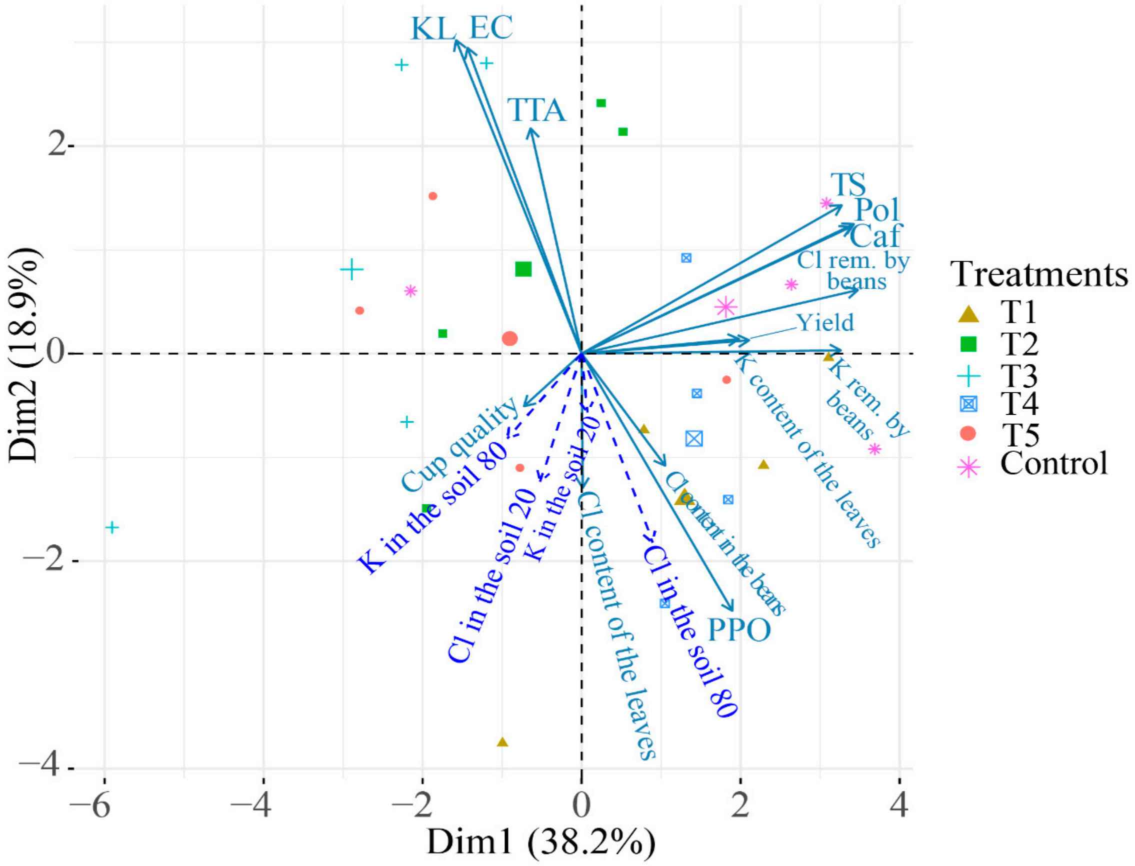

Figure S8. Principal component analysis for the harvest of 2017/2018. PPO = activity of the enzyme polyphenol oxidase; KL = K leaching; TTA = total titratable acidity; EC = electric conductivity; Pol = total phenolic compounds; Caf = content of caffeine; TS = content of total sugars; K in the soil 20 = stock of K in the 0-20 cm layer; K in the soil 80 = stock of K in the 20-80 cm layer; Cl in the soil 20 = stock of Cl in the 0-20 cm layer; Cl in the soil 80 = stock of Cl in the 20-80 cm layer, K rem. by beans: K removal by the beans, Cl rem. by beans: Cl removal by the beans. T1: 100% KCl; T2: 75% KCl + 25% K_2SO_4; T3: 50% KCl + 50% K_2SO_4; T4: 25% KCl + 75% K_2SO_4; T5: 100% K_2SO_4; control did not receive K_2O.
